# Growth Mindset and College Students’ Learning Engagement During the COVID-19 Pandemic: A Serial Mediation Model

**DOI:** 10.3389/fpsyg.2021.621094

**Published:** 2021-02-19

**Authors:** Hui Zhao, Jianping Xiong, Zhen Zhang, Chunhui Qi

**Affiliations:** Faculty of Education, Henan Normal University, Xinxiang, China

**Keywords:** learning engagement, growth mindset, perceived COVID-19 event strength, perceived stress, COVID-19 pandemic

## Abstract

Against the scourge of the COVID-19 pandemic, college students’ learning engagement has become a key issue in universities and society. Guided by the theories of existential positive psychology and social perception, we explored the positive effect of a growth mindset on learning engagement during the COVID-19 pandemic. A total of 1,040 college students from universities in Henan Province of China effectively completed online questionnaires. The results showed that growth mindset was positively related to learning engagement and negatively associated with perceived COVID-19 event strength and perceived stress; perceived COVID-19 event strength was positively related to perceived stress, while perceived COVID-19 event strength and perceived stress were negatively associated with learning engagement. Growth mindset affected learning engagement through three indirect paths: the mediating role of perceived COVID-19 event strength, the mediating role of perceived stress, and the serial mediating role of both perceived COVID-19 event strength and perceived stress. The results indicated that the growth mindset could contribute to college students’ learning engagement through the roles of perceived COVID-19 event strength and perceived stress during the COVID-19 pandemic. This study advances the understanding of the mechanism underlying the relationship between growth mindset and college students’ learning engagement during the COVID-19 pandemic. Furthermore, the findings of the study have important implications for promoting college students’ learning engagement during the pandemic.

## Introduction

Due to the spread of the COVID-19 pandemic, physical distancing procedure was employed in China. During the months of social isolation and movement restrictions, the normal order of teaching had been disrupted. Guided by the policy of “Classes suspended but learning continues” (Jiao et al., 2020), Chinese colleges and universities guaranteed the progress of teaching and the quality of higher education through various network platforms and ultimately created a new teaching model that combined online teaching and autonomous learning. Although China’s pandemic prevention and control work is generally recognized, problems such as lower levels of student engagement are inevitable (Yang et al., 2020). Thus, exploring the factors influencing students’ learning engagement to ensure the effective teaching during the pandemic becomes especially important. As a reflection of the positive state of the learning process, learning engagement is defined as a continuous and positive emotional state while learning (Schaufeli et al., 2002). It is an important factor that affects the quality of learning. For example, learning engagement has a positive effect on learners’ learning performance and completion of learning tasks (Pursel et al., 2016; Bergdahl et al., 2020). From existential positive psychology (PP 2.0), a meaningful state depends on embracing and transforming suffering (Wong, 2019). This means that we need to actively confront the dark sides of human existence such as the COVID-19 pandemic to maintain a positive learning state. Considering the important role of college students’ learning engagement and the negative impact of the COVID-19 pandemic, this study focuses on the factors affecting learning engagement during the pandemic.

Growth mindset refers to an individual’s implicit cognitive belief about the variable degree of his or her basic traits (e.g., intelligence, ability) (Dweck, 2006). Someone has a growth mindset who believes that people have differences in traits, but their traits can be constantly changed and developed through unremitting efforts and are not “carved in stone” (Dweck, 2006). The PP 2.0 emphasizes flourishing through suffering (Wong, 2020). Researcher advocated that the best way to cope with suffering and existential crisis is to obtain a mindset, which includes self-transcendence and self-actualization (Frankl, 2011). However, the vast literatures on growth mindset can be interpreted in terms of the basic human motivation for self-transcendence (Wong, 2012). Numerous studies have indicated that the growth mindset is significantly and positively linked with students’ learning engagement (Lin-Siegler et al., 2016; Schmidt et al., 2017). That is, students with high levels of growth mindset may achieve higher learning engagement. However, the relationship between growth mindset and learning engagement has not been tested among college students during the pandemic. Moreover, the internal mechanism of this relationship remains unclear. Therefore, this study replicated the association between growth mindset and learning engagement by using a sample of isolated Chinese college students during the COVID-19 pandemic. Furthermore, we examined the internal mechanism of above relationship.

## Growth Mindset and Learning Engagement

Coping with an existential crisis, the PP 2.0 emphasizes uniquely human mindsets and capacities for positive change (Wong, 2010). Social perception theory proposes that people judge, interpret, and process information in environment through implicit theory and show different feelings and behaviors (Heider, 1958). As a positive implicit theory, growth mindset that implies the motivation for self-transcendence may become an effective force for coping with the COVID-19 pandemic and increasing students’ learning engagement. This study introduced growth mindset into the pandemic context and investigated the relationship between growth mindset and learning engagement.

Research on engagement has proposed that the historic challenges of the COVID-19 pandemic demands growth mindset (Risley, 2020). Dweck believed that the awareness of taking risks and challenges, and working hard stems directly from people’s basic growth mindset (Dweck, 2006). Study has indicated that in adverse or challenging situations, the higher the level of growth mindset that people hold, the more value they will perceive in challenges, and they tend to invest more time and effort to continuously improve their situation (Liu et al., 2014). Previous studies in the field of education have shown that growth mindset is positively related to academic self-efficacy and academic performance (Diao et al., 2020). Growth mindset cannot only increase students’ effort, energy, and perseverance to complete learning tasks but also encourage learners to achieve higher academic achievement (Yeager et al., 2019). Growth mindset is a known predictor of academic achievement, as students characterized by it try new strategies and seek assistance when needed, in addition to exerting more effort (Claro et al., 2016). It can be inferred that students’ learning is related to growth mindset. Specific research on learning engagement has indicated that growth mindset intervention can change students’ motivational beliefs, thereby increasing their learning engagement (Lin-Siegler et al., 2016). Moreover, growth mindset can also improve students’ sense of control, stimulate their interest and expectancy in learning, and help them maintain learning engagement (Schmidt et al., 2017). Therefore, based on previous evidence, we propose the following hypothesis in the pandemic:

Hypothesis 1: Growth mindset has a positive effect on learning engagement.

## The Mediating Roles of Perceived COVID-19 Event Strength and Perceived Stress

Social perception theory proposes that implicit theory provides people with a mental model that guides them to think, feel, and act in challenging situations (Heider, 1958). PP 2.0 claims that it is necessary to understand, accept, and embrace with courage the reality that life is full of suffering to bring out the best in people (Wong, 2010). Does this mean that during a COVID-19 event, a student’s growth mindset affects his or her perception and feeling, which acts on their learning status? Perceived COVID-19 event strength is a perception of external events, while perceived stress is a feeling of internal pressure. Their unique roles during the pandemic have attracted our attention.

The event system theory proposes that the strength of an event determines the impact the event has on an individual (Morgeson et al., 2015). Event strength includes novelty, disruption, and criticality of an event (Liu and Liu, 2017). From the perspective of cognitive psychology, the importance of events is generated in the mind of the person facing the event. Perceived COVID-19 event strength refers to an individual’s subjective evaluation of the strength of the COVID-19 pandemic event. Growth mindset that affected students’ responses to adversity was found (Wang and Amemiya, 2019). That is, when facing external challenges or setbacks, individuals with growth mindsets adopt novel strategies and self-regulation to overcome difficulties (Kench et al., 2016). A positive mindset means that they no longer regard suffering caused by COVID-19 as a dreadful enemy, but as a warning that their life is out of balance and a signal for them to overcome their setbacks and find a new path of meaning and purpose (Wong, 2020). Students with growth mindset show a high ability to resist negative events (Wang and Amemiya, 2019). To courageously face and flexibly respond to COVID-19 contexts, students may use their growth mindset to adjust cognition and reduce their perceptions of COVID-19 event strength. Furthermore, individuals with high awareness of negative events cannot effectively regulate and resolve negative emotions, which have a positive predictive effect on learning burnout in college students (Liu et al., 2019). Students who are better able to face adverse events have higher levels of academic performance (Hojat et al., 2003). Thus, the following hypothesis is developed in relation to the pandemic:

Hypothesis 2: Perceived COVID-19 event strength mediates the relationship between growth mindset and learning engagement.

Perceived stress refers to feelings of tension, threat, and so forth, which is caused by negative events or adverse factors (Yan et al., 2010). It is a subjective feeling of pressure. Improvements in growth mindset can decrease academic stress and worry (Yeager et al., 2016). The latest research has indicated that growth mindset can buffer academic stress faced during the COVID-19 pandemic related to social isolation (Mosanya, 2020). The theoretical framework proposed by Burnette et al. (2013) suggested that such an effective impact is due to the mindset influence on self-regulatory processes. Jegathesan et al. (2016) also found that individuals with higher growth mindset improved their emotions through positive coping. The above shows that growth mindset may negatively predict perceived stress. Moreover, the perception of stress has always been regarded as a key factor affecting individuals’ learning motivation, learning engagement, and academic performance (Pascoe et al., 2020). It is demonstrated that negative emotional states brought about by higher perceptions of stress can predict poor academic performance in students (Kötter et al., 2017). Furthermore, perceived stress has a negative effect on students’ internal learning motivation (Liu Y., 2015). In the study of learning engagement, negative emotional experiences are directly related to lower learning engagement among students (Reschly et al., 2008). Based on these findings, it can be inferred that perceived stress may decrease students’ learning engagement. Thus, we propose the following:

Hypothesis 3: Perceived stress mediates the relationship between growth mindset and learning engagement.

In summary, the perceived COVID-19 event strength and perceived stress are important links that connect growth mindset and learning engagement. Different types of negative life events are specifically related to mental health problems (including stress) (Liu et al., 2019). Research also directly pointed out that negative life events can cause perceived stress (Zhang et al., 2016). Perceived COVID-19 event strength may be positively related to perceived stress. Individuals with growth mindset would better adapt to a stressful environment, and improved their emotions through positive coping (Jegathesan et al., 2016). If college students experiencing negative events cannot effectively regulate their emotions, their learning status will be significantly affected (Liu et al., 2019). Based on the above, students with growth mindset tended to better adapt to events and have a lower perception of event strength, which further weakened perceived stress and enhanced their learning engagement. Thus, the following hypothesis is proposed:

Hypothesis 4: Growth mindset can affect learning engagement through the serial mediating roles of perceived COVID-19 event strength and perceived stress.

**FIGURE 1:**
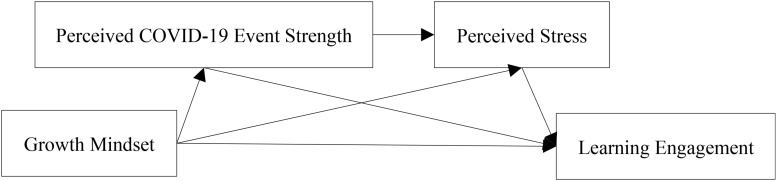
Research model.

Based on the theories of PP 2.0 and social perception, this study aimed to test the positive impact of a growth mindset on learning engagement in isolated college students during the COVID-19 pandemic. Furthermore, this study explored the internal mechanism of growth mindset affecting learning engagement, which is, examining the mediating roles of perceived COVID-19 event strength and perceived stress. This study provided a theoretical reference for the improvement of college students’ learning engagement during the pandemic. Figure 1 depicts the research model.

## Materials and Methods

### Participants and Procedure

During the period of the COVID-19 pandemic (February to March 2020) in China, we were limited by China’s home isolation policy; thus, we used an online questionnaire survey platform^1^ to collect data. To reduce the dropout rates of the survey, the study chose the short form of questionnaires to measure the research variables. The participant could submit the questionnaire only after all the items had been completed to reduce the possibility of accidentally skipping items. According to the logged-in WeChat account, one participant was only allowed to submit reply once. Since the self-report questionnaires might be affected by participants’ response bias, all participants remained anonymous and participated voluntarily. Furthermore, participants were informed in the instruction section of the questionnaire that, “This study is purely a scientific investigation and has nothing to do with your academic performance and evaluation. There are no good or bad answers, but through data analysis we can identify whether you have cheated, so please be sure to fill in objectively and truthfully.” Participants were also told that if they felt uncomfortable, they were free to withdraw from the survey halfway.

Henan Province is adjacent to Hubei Province where Wuhan City is located. Affected by the pandemic of Wuhan City, Henan Province became one of the key pandemic areas in China in the early stages of the pandemic. Restricted by isolation policy and movement restrictions, the questionnaire was sent to familiar teachers from universities in Henan Province of China *via* WeChat and email. Then, with the help of teachers, the questionnaire was sent to students’ WeChat talking groups. Finally, a total of 1,076 questionnaires were collected. If all questions had the same answers, the choices of answers were extremely regular, if the answers to the questions were contradictory, and answering time was less or more than the normal value, these questionnaires were excluded. A total of 1,040 valid questionnaires were retained. The demographic survey results showed that participants comprised 396 males (38.1%) and 644 females (61.9%). The ages ranged from 18 to 24 years. There were 280 freshmen (26.9%), 451 sophomores (43.4%), 200 juniors (19.2%), and 109 seniors (10.5%). There were 657 rural students (63.2%) and 383 urban students (36.8%). Our study was conducted according to the recommendations of the Ethics Committee of Henan Normal University.

### Measures

Growth mindset was evaluated using the Chinese version of Dweck Mindset Instrument (Dweck et al., 1995). The Chinese version of this scale showed good reliability when used to measure college students’ mindsets in China (e.g., Lei et al., 2017; Ma et al., 2020). This scale had three items (e.g., “You have a certain amount of intelligence and you really can’t do much to change it”). Items were rated on a 6-point Likert scale (1 = strongly agree, 6 = strongly disagree). The mean score of three items was calculated, with higher scores showing the stronger growth mindset. Cronbach’s alpha coefficient for the scale was 0.85.

Perceived COVID-19 event strength was assessed using the Chinese version of Event Strength Scale developed by Morgeson (2005) and translated into Chinese by Liu and Liu (2017). The Chinese version of this scale has been widely adopted in prior Chinese researches and has been found to have high reliability (e.g., Xu and Chen, 2020). The scale had a total of 11 items, including three subscales: event novelty (e.g., “There is a clear, known way to respond to the COVID-19 event”), event criticality (e.g., “COVID-19 event is critical for the long-term success of me”), and event disruption (e.g., “COVID-19 event disrupts my ability to get its learning done”). The items in the event novelty subscale were reverse scored. Items were rated on a 5-point Likert scale (1 = not at all, 5 = to a very large extent). For each item, college students were required to choose the extent to which these descriptions fit their actual situation. Cronbach’s alpha coefficient for the scale was 0.70.

Perceived stress was evaluated using the Chinese Perceived Stress Scales (Yang and Huang, 2003). The scale showed high reliability and was widely used to measure perceived stress in Chinese research (e.g., Li and Ma, 2019; Fan and Yuan, 2020). The scale had a total of 14 items, including two subscales: tension (e.g., “feeling nervous and stressed”) and a sense of loss of control (e.g., “feeling unable to control the important things in your life”). Items were rated on a 5-point Likert scale (1 = strongly disagree, 5 = strongly agree). The score was the average of the applicable items, with larger values indicating a greater perceived stress. Cronbach’s alpha coefficient for the scale was 0.83.

Learning engagement was assessed using the Chinese version of 9-item Utrecht Work Engagement Scale (Schaufeli et al., 2006). The Chinese version of this scale has been validated to measure learning engagement among Chinese participants and has been found to have good psychometric properties (e.g., Zhou, 2018). The scale included three subscales: vigor (e.g., “At my work, I feel bursting with energy”), dedication (e.g., “I find the work that I do full of meaning and purpose”) and absorption (e.g., “Time flies when I am working”). Items were rated on a five-point Likert scale (1 = strongly disagree, 5 = strongly agree). Cronbach’s alpha for scale reliability was 0.91.

To exclude the influence of demographic characteristics on the research results, gender (male or female) and birthplace (urban or rural) were included as control variables. Gender and urban-rural factors had a greater impact on college students’ learning engagement than other demographic characteristics (e.g., disciplines and socio-economic status) (Yang and Zhang, 2016). Specifically, females had a higher level of learning engagement than males (Wen et al., 2010; Yang and Zhang, 2016). Furthermore, there were significant differences in the development of students’ learning engagement in urban and rural areas (Liu Z., 2015). Thus, gender and birthplace were controlled in the data analysis process.

### Data Analysis

In the common-method bias test, Harman’s single-factor test was performed using SPSS 23.0, and the method-factor approach was performed using Amos 23.0. SPSS 23.0 was also selected for the multicollinearity test, reliability analysis, and correlation analysis of variables. Finally, hypotheses were tested with the PROCESS macro of SPSS (Hayes, 2013; Model 6, 5,000 bootstrap resamples).

## Results

### Multicollinearity Test and Common-Method Bias Test

Before data analysis, the original questionnaire data needed to be processed. First, we conducted a multicolinearity test and found that the tolerance range of each variable was between 0.86 and 0.93 (all greater than 0.1), and the variance expansion factor ranged between 1.08 and 1.16 (all less than 10). These findings showed that there was no multicolinearity problem among the variables in this study.

Harman’s single-factor test (Eby and Dobbins, 1997) and the method-factor approach (Xiong et al., 2012) were used to statistically verify the presence of common method bias. The results of Harman’s single-factor examination showed that eight factors had eigenvalues greater than one, and the first factor explained 22.52% of the total variance. This result did not exceed the critical value of 40%. Furthermore, the confirmatory factor analysis (CFA) results of the method-factor approach showed that the model fit was not significantly improved after adding the common method factor (χ*^2^/*df** = 3.738, *CFI* = 0.914, *IFI* = 0.914, *RMSEA* = 0.051) to the four-factor model of this study (χ*^2^/*df** = 3.740, *CFI* = 0.913, *IFI* = 0.914, *RMSEA* = 0.051). The above methods demonstrated that serious common method bias did not exist.

### Correlations Between Primary Variables

The Spearman correlations presented in Table 1 showed that there were significant positive relationships between growth mindset and learning engagement as well as between perceived COVID-19 event strength and perceived stress. There were significant negative relationships between growth mindset and perceived COVID-19 event strength, perceived COVID-19 event strength and learning engagement, growth mindset and perceived stress, and perceived stress and learning engagement. The table showed that growth mindset, perceived COVID-19 event strength, perceived stress, and learning engagement were closely related. The correlation coefficients were moderate (far lower than Cronbach’s alphas). These findings met the prerequisites for conducting hypothesis testing.

**TABLE 1:** Means, standard deviations, correlations, and reliabilities (in brackets).

	*M*	*SD*	1	2	3	4	5	6
1 Gender^a^	0.38	0.49	−					
2 Birthplace^b^	0.37	0.48	0.01	−				
3 GM	3.28	0.96	0.01	0.01	(0.85)			
4 PCES	2.80	0.43	–0.03	−0.15**	−0.20**	(0.70)		
5 PS	2.60	0.49	–0.02	−0.08*	−0.23**	0.33**	(0.83)	
6 LE	3.29	0.60	0.01	0.10**	0.13**	−0.26**	−0.39**	(0.91)

*p < 0.05, **p < 0.01, *N* = 1,040 for college students, gender ^*a*^(“0” female; “1” male), birthplace ^*b*^(“0” urban; “1” rural); GM, growth mindset; PCES, perceived COVID-19 event strength; PS, perceived stress; LE, learning engagement. The numbers in brackets on the diagonal line are the Cronbach’s alphas.

### Hypothesis Testing

We conducted a serial mediation model with gender and birthplace as the control variables, growth mindset as the independent variable, and perceived COVID-19 event strength and perceived stress as mediators of the effect of learning engagement.

Table 2 showed that growth mindset has a significant and positive effect on learning engagement. In the path of “growth mindset → perceived COVID-19 event strength → learning engagement,” growth mindset had a significant negative impact on perceived COVID-19 event strength (β = −0.09, *p* < 0.001), while perceived COVID-19 event strength had a significant negative impact on learning engagement (β = −0.18, *p* < 0.001). Thus, growth mindset enhanced learning engagement by reducing perceived COVID-19 event strength. In the path of “growth mindset → perceived stress → learning engagement,” growth mindset had a significant negative impact on perceived stress (β = −0.09, *p* < 0.001), while perceived stress had a significant negative impact on learning engagement (β = −0.42, *p* < 0.001). Thus, growth mindset improved learning engagement by weakening students’ perception of stress. In the path of “growth mindset → perceived COVID-19 event strength → perceived stress → learning engagement,” perceived COVID-19 event strength had a significant positive impact on perceived stress (β = 0.33, *p* < 0.001). This indicates that the perceived COVID-19 event strength was closely related to students’ perceived stress. Furthermore, growth mindset reduced the perception of stress by alleviating the perceived COVID-19 event strength, which increased students’ learning engagement. These results supported hypotheses 1–4.

**TABLE 2:** Model results.

Variables	Model 1			Model 2			Model 3		
	PCES			PS			LE		
	β	*SE*	*t*	β	*SE*	*t*	β	*SE*	*t*
Constant	3.14	0.05	65.83***	1.98	0.12	16.60***	4.80	0.16	29.72***
Gender^a^	–0.02	0.03	–0.84	–0.02	0.03	–0.517	0.00	0.04	0.05
Birthplace^b^	–0.13	0.03	−4.92***	–0.03	0.03	–1.01	0.07	0.04	1.84
GM	–0.09	0.01	−6.42***	–0.09	0.02	−5.97***	0.02	0.02	1.00
PCES				0.33	0.03	9.63***	–0.18	0.04	−4.26***
PS							–0.42	0.04	−11.14***
*R*^2^		0.06			0.14			0.18	
*F*		22.27***			41.11***			43.83***	

*p < 0.05, **p < 0.01, ***p < 0.001, *N* = 1,040 for college students, gender ^*a*^(“0” female; “1” male), birthplace ^*b*^(“0” urban; “1” rural); GM, growth mindset; PCES, perceived COVID-19 event strength; PS, perceived stress; LE, learning engagement.

**FIGURE 2:**
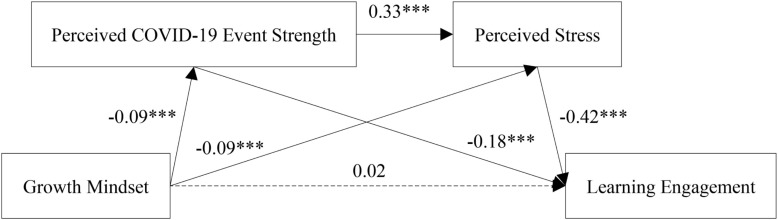
Roadmap of the influence of growth mindset on learning engagement.

Furthermore, Table 2 (Model 3) showed that the direct effect of growth mindset on learning engagement was not significant (β = 0.02, *p* > 0.05). Perceived COVID-19 event strength and perceived stress had a full mediating effect on the relationship between growth mindset and learning engagement (see Figure 2).

Following above tests, we further observed the mediating effects of perceived COVID-19 event strength and perceived stress on the relationship between growth mindset and learning engagement. Consistent with our findings, Table 3 showed that the total indirect effect of perceived COVID-19 event strength and perceived stress is 0.07; thus, perceived COVID-19 event strength and perceived stress had a significant mediating effect on the relationship between growth mindset and learning engagement. Specifically, the mediating effect was composed of indirect effects produced by three paths: the mediating role of perceived COVID-19 event strength [95% confidence interval (CI), 0.01–0.03], the mediating role of perceived stress (95% CI, 0.02–0.05), and the serial mediating role of both perceived COVID-19 event strength and perceived stress (95% CI, 0.01–0.02). All three indirect effect paths were significant. Furthermore, the path of “growth mindset → perceived stress → learning engagement” had the strongest mediating effect. A comparison of the three paths of mediation showed that growth mindset mainly improved learning engagement by reducing perceived stress.

**TABLE 3:** Effects and 95% confidence intervals for Model 3.

Growth mindset	Effect	Boot SE	Boot LLCI	Boot ULCI
Direct effects	–0.02	0.02	–0.02	0.05
Total indirect effect	0.07	0.01	0.05	0.09
Indirect effect 1	0.02	0.01	0.01	0.03
Indirect effect 2	0.04	0.01	0.02	0.05
Indirect effect 3	0.01	0.00	0.01	0.02

*The path of indirect effect 1 is “growth mindset → perceived COVID-19 event strength → learning engagement,” the path of indirect effect 2 is “growth mindset → perceived stress → learning engagement,” and the path of indirect effect 3 is “growth mindset → perceived COVID-19 event strength →perceived stress → learning engagement.”*

## Discussion

What remains to be answered is whether growth mindset plays a positive role in learning engagement during the pandemic period and how growth mindset contributes to learning engagement. Thus, the current study explored the relationship between growth mindset and learning engagement. Furthermore, the mediating roles of perceived COVID-19 event strength and perceived stress in the above relationship were examined among isolated Chinese college students. Our findings have showed that growth mindset has an indirect and positive effect on learning engagement. Additionally, perceived COVID-19 event strength and perceived stress, respectively, mediated the relationship between growth mindset and learning engagement. Moreover, serial mediation existed among those variables: students with growth mindset tended to report lower perceived COVID-19 event strength, which further decreased their perceived stress levels and, subsequently, promoted their learning engagement.

### Theoretical Implications

This study has discovered that in the context of the pandemic, growth mindset has an indirect and positive effect on learning engagement. This study further revealed the effects of growth mindset from the perspective of PP 2.0 and answered the theoretical question of whether growth mindset affects learning engagement in the pandemic. A positive correlation between growth mindset and learning engagement in the context of the COVID-19 pandemic was found, which is consistent with the results of an intervention study showing that growth mindset can help individuals maintain learning engagement in non-pandemic contexts (Schmidt et al., 2017). Therefore, the growth mindset influenced learning engagement in the COVID-19 pandemic context as well as in other contexts. One potential explanation for this finding of the present study is that growth mindset implies the motivation for self-transcendence (Wong, 2012; Howell, 2016). Individuals with growth mindsets adopt learning goal orientation, which is positively correlated with self-transcendence values (Pudelko and Boon, 2014). Meanwhile, the motivation for self-transcendence help individuals to obtain higher levels of meaning, such as the realization of personal potential (Zhang et al., 2020) and the improvement of engagement (Palmer et al., 2010). In addition, growth mindset is also related to grit, helping students persist in adverse circumstances (Tuckwiller and Dardick, 2018). Individuals with growth mindsets show consistent interests in learning tasks, and exhibit persistent efforts to complete these tasks in adversity. Life consists of various diseases and pain, but this does not stop people with growth mindset from determination of self-transcendence and continuing their efforts. Based on the above reasons, students with growth mindsets can engage in learning even in the pandemic. The results of this study provide empirical support for PP 2.0 and further affirm the positive significance of growth mindset to learning engagement in the pandemic.

This study discovered the mediating roles of perceived COVID-19 event strength and perceived stress between growth mindset and learning engagement. It also found that serial mediation existed in the above relations in the context of the pandemic. The findings again support that it is people’s cognitive beliefs that enable them to overcome negative conditions and explore negative experiences by themselves, regardless of a harsh situation (Wong, 2011; Zhang et al., 2020). The internal mechanism underlying the relationship between growth mindset and learning engagement in the COVID-19 pandemic was revealed. These findings further enrich the literatures on the individual mental world under suffering and the existential crisis. The potential reasons for the findings are as follows: Implicit beliefs generally affect the meaning with which experiences are imbued, which represents understanding of meaning (Howell, 2016). The growth mindset can be seen as positive internal “code” or belief, shaping people’s understanding of the COVID-19 event strength. Moreover, Mindset may foster self-transcendence (Seligman, 2011). A meaning mindset with self-transcendence values can remain positive and hopeful, even if everything seems bleak (Wong, 2016). Self-transcendence is related to finding meaning and purpose in life, being optimistic about the future, and coping with difficulties, which allows individuals to accept the current life situations and stressors more readily (Reed, 2014; Morrison, 2018). Therefore, students with growth mindsets could better adapt to the COVID-19 event and experience lower perceived stress. At last, growth mindset promoted resilience when faced with challenges (Blackwell et al., 2007; Yeager and Dweck, 2012). Students with growth mindsets are good at seeking adaptive strategies to deal with external challenges and then regulating their emotions (Cooley and Suzanne, 2018). Therefore, they with growth mindsets had lower perceived COVID-19 event strength and perceived stress, which kept them in continuous and active learning states. This serial mediation model strongly explains the intermediary mechanism through which growth mindset influences learning engagement.

This study focused on Chinese college students during the COVID-19 pandemic. The relationship between growth mindset and learning engagement was examined in the Chinese culture and particular context of the COVID-19 pandemic. The existing research results showed that there were differences in psychological and learning engagement among individuals with different learning stages, occupational fields, and cultural backgrounds (Martin, 2009; Martin et al., 2014; Yin, 2020). However, previous studies mainly explored the role of growth mindset on engagement in other groups such as employees (e.g., Risley, 2020) and learning engagement in other countries such as the United States (e.g., Lin-Siegler et al., 2016; Schmidt et al., 2017), but few have focused on college students and Chinese culture. This study enriches the body of knowledge from previous research and paves the way for future studies on learning engagement and other learning variables in different groups and different cultures. Furthermore, the ongoing outbreak of COVID-19 is rapidly spreading globally (Liu et al., 2020). The results of affirming the positive significance of the growth mindset in the pandemic support the conclusions of previous studies in non-pandemic contexts. This study provides a theoretical reference for the education of other countries in the context of the COVID-19 pandemic.

### Practical Implications

Colleges and universities should strengthen the shaping of students’ growth mindset to increase students’ learning engagement. First, course training has been widely used in intervention research on growth mindset (Blackwell et al., 2007; O’Brien and Lomas, 2017). During the pandemic, the growth mindset intervention targeted at college students can be carried out through online course training. In course training, teachers can use scientific reading, activities, and discussions to shape students’ cognitive beliefs that abilities can be changed and developed, and constantly encourage students to work hard and overcome difficulties. When students successfully overcome difficulties and make major progress through independent efforts, teachers should provide timely and appropriate encouragement to continuously improve their growth mindset. Second, growth mindset can be changed through letter exchange interventions (Good et al., 2003). This is an intervention method that teaches growth mindsets through communication between intervening personnel and students. During the pandemic, psychology teachers can provide psychological interventions for students through emails to subtly improve students’ growth mindset and increase their learning engagement. Third, autonomy support is positively associated with growth mindset (Ma et al., 2020). Thus, during the COVID-19 pandemic or other special periods, colleges and universities can formulate flexible learning plans for students as appropriate. This approach can encourage students to formulate micro-learning plans according to their own circumstances and needs, and enhance students’ self-transcendence through independent analysis, exploration, and practice.

Colleges and universities should actively respond to the pandemic and ease students’ perceived event strength to maintain students’ learning engagement. First, teachers should encourage students to reinterpret the COVID-19 pandemic and other surrounding events with the belief of self-transcendence, and devote themselves to learning in a full and pleasant way. Once we are awakened to transcendental values, we become truly alive and fearless (Wong, 2020). This can enable students to form appropriate control tendencies, actively cope with external challenges, and reduce the impact that the pandemic has on them. Second, the study found that during the pandemic period, full and in-depth teaching interaction could enable students to feel the “scaffolding” role of teachers throughout the learning process and enter the development track of “facing challenges–gradually competent–gaining recognition” (Ma, 2020). Teachers should provide timely online support and guidance and give quick feedback to homework. A variety of online information platforms providing different functions should also be used to buffer the negative impact of the pandemic on learning engagement. Third, during crisis events, colleges and universities should pay attention to the positive effects of role models on college students’ perception of the event strength. Based on pp 2.0, people need to discover something beautiful in life, no matter how brutal life is (Wong, 2020). Colleges and universities must actively promote the efforts of frontline doctors, soldiers, and ordinary people in the fight against the pandemic (Song and Xu, 2020). Role models from student volunteers around them can also be used to encourage students.

Colleges and universities should reduce students’ perceived stress to promote students’ learning engagement. Wong et al. (2006) proposed a resource-congruence model of effective coping. The model points out that when an individual is faced with a stressful event, if there are enough internal and external resources, the stress experienced by the individual will be relieved. In addition to internal resources such as a growth mindset, the effective factors for students to reduce their perceived stress also include external resources such as intimate interpersonal relationships and fair education evaluation. First, in terms of interpersonal relationships, the situation of students as “lone learners” may cause perceived stress. Teachers can use online teaching to eliminate the shackles of traditional teaching methods and promote the development of the “teacher-parent-student” community (Song et al., 2020). Teachers should discuss curriculum arrangements with parents and students and consciously design cooperative and interactive learning activities, while creating a virtual classroom community to address students’ physical separation. These measures can promote student–teacher, student–student, student–parent, and teacher–parent relationships. Second, in terms of education evaluation, colleges and universities should change traditional education evaluation methods to relieve academic pressure caused by grades. In addition to traditional course tests, online platform tracking data can also be incorporated into student performance information, along with information from parent observations and data regarding students’ enthusiasm, participation, collaboration, and task completion.

## Limitations and Future Research

This study explored the mechanism underlying growth mindset’s impact on learning engagement during the COVID-19 pandemic. Although it has certain theoretical and practical implications, it also has several limitations. First, limited by China’s home isolation policy, it was difficult for us to conduct a questionnaire survey among college students across the country, including Wuhan in the early stages of the pandemic. In the face of different crisis events in the future, the scope of investigation should be further expanded. Second, the data was collected through student self-reports and the students were recruited by their teachers. So, this data might be affected by participants’ response bias. In the future, data from teacher evaluations and parent observations can be added for comprehensive measurement. Third, this study revealed the internal mechanism from the perspectives of cognitive and emotional factors. Future research must further consider whether there are other intermediary pathways underlying the influence of growth mindset on learning engagement, such as motivation. Furthermore, researchers emphasized that when studying students’ learning engagement, not only the internal psychological states and external engagement behaviors must be considered at the same time, but also the socio-cultural context (Martin et al., 2014; Yin, 2020). We need to further study whether the socio-cultural context can potentially enhance or inhibit the positive effects of growth mindset on college students’ learning engagement.

## Conclusion

The study from the perspective of PP 2.0 highlighted the importance of the growth mindset in promoting college students’ learning engagement in the context of the COVID-19 pandemic. Moreover, the findings showed that facing the COVID-19 pandemic, students with growth mindset had lower event strength and perceived stress, which encouraged them to maintain a higher level of learning engagement. It is hoped that this study will stimulate further research and discussion concerning growth mindset or learning engagement.

## Data Availability Statement

The raw data supporting the conclusions of this article will be made available by the authors, without undue reservation.

## Ethics Statement

The studies involving human participants were reviewed and approved by Ethics Committee of Henan Normal University. The patients/participants provided their written informed consent to participate in this study.

## Author Contributions

HZ conceived the research idea, and structured and drafted the manuscript. JX, ZZ, and CQ collected and analyzed the data. All authors contributed to the article and approved the submitted version.

## Conflict of Interest

The authors declare that the research was conducted in the absence of any commercial or financial relationships that could be construed as a potential conflict of interest.

## Footnotes

1 www.wjx.cn
